# Interpretable Spectral Evidence Learning from Vis/NIR Imaging for Non-Destructive Authentication of Herbal Medicines

**DOI:** 10.3390/molecules31142444

**Published:** 2026-07-12

**Authors:** Zhihui Fan, Chao Ma, Shaowen Jing, Jiayu Huang, Mingkun Zhang

**Affiliations:** 1 College of Information Engineering, Henan University of Science and Technology, Luoyang 471023, China; fzh@haust.edu.cn (Z.F.); 9906066@haust.edu.cn (C.M.);; 2 College of Electronics and Information Engineering, South China University of Technology, Guangzhou 510641, China

**Keywords:** visible and near-infrared imaging, herbal authentication, lightweight diffusion, spectral augmentation, wavelength evidence, non-destructive detection

## Abstract

Rapid and non-destructive authentication of herbal medicines is important for quality control and market supervision. This study established an interpretable spectral evidence learning framework for visible and near-infrared (Vis/NIR) imaging-based authentication of Codonopsis Radix (CR) and Aurantii Fructus (AF). Compact 31-band mean gray-value spectra were analyzed at ROI and sample levels. CR sample-level spectra were obtained by ROI-group averaging, whereas AF records were retained as individual sample spectra with image-group information used for leakage-controlled validation. Raw spectra, Savitzky–Golay smoothing, multiplicative scatter correction, and standard normal variate correction were compared with machine-learning and deep-learning classifiers. A fold-contained lightweight diffusion (LD) module was further introduced to provide class-conditioned spectral augmentation and denoising-error evidence. Under grouped cross-validation, the strongest non-LD Linear SVM models achieved accuracy/macro-F1 values of 0.9231/0.9238 for CR and 0.9025/0.9018 for AF. After LD augmentation, the best LD-augmented SVM models reached macro-F1 values of 0.9427 and 0.9197, respectively. Across all evaluated model–dataset combinations, LD increased the overall mean macro-F1 from 0.7302 to 0.8189. Model-aligned wavelength evidence and top-wavelength subset tests further showed that selected LED-band subsets retained useful discriminative information within the present imaging configuration. These results support the feasibility of compact Vis/NIR image-based authentication of herbal materials under grouped validation.

## 1. Introduction

Herbal medicines and food-related medicinal materials require reliable quality control because substitution, mislabeling, and origin-related variability can affect market supervision and consumer confidence. Recent reviews emphasize that visible/near-infrared spectroscopy and hyperspectral imaging can provide rapid, non-destructive analytical evidence for food and natural-product inspection [1,2,3]. Vis/NIR and NIR workflows have recently been used for counterfeit Citri Reticulatae Pericarpium identification, turmeric adulteration detection, and herbal-tea adulteration quantification [4,5,6]. Related authentication studies on paprika, argan oil, and apricot kernels further show that chemometric models can support authentication when applied to compact spectral datasets [7,8,9].

The present work focuses on Codonopsis Radix (CR) and Aurantii Fructus (AF). The CR dataset included authentic CR and three visually related substitute classes: Sphallerocarpus gracilis (SGR), Saposhnikoviae Radix (SR), and Platycodonis Radix (PR). Recent CR studies have examined digital chemical identity, machine-learning quality assessment, and electronic-nose prediction of chemical and sensory information [10,11,12]. Studies on citrus fruit have also shown that Vis/NIR spectral imaging combined with machine-learning models can support counterfeit identification and geographical-origin authentication of food–medicine homologous materials [13,14,15]. Additional Codonopsis work has used odor information and metabolomics to describe origin-related and tissue-related chemical variation [16,17]. The AF dataset included four production origins: Jiangxi–Yichun, Hunan–Yiyang, Sichuan–Meishan, and Chongqing–Jiangjin. Recent AF studies have reported metabolomic quality traits, chemometric comparison with Aurantii Fructus Immaturus, and UHPLC-Q-TOF-MS/MS quality evaluation [18,19,20]. AF-related chemical imaging and network-pharmacology studies provide complementary chemical and biological context for understanding Aurantii Fructus. However, the spectral classification results in the present study should be interpreted as optical evidence associated with chemical and physicochemical differences, rather than as direct compound-level quantification or pharmacological evidence [21,22]. Related studies on food–medicine homologous substances and traditional medicinal materials motivate the use of portable optical and sensory screening, but literature records are not used as substitutes for local sample provenance and voucher information [23,24,25].

Despite these advances, several limitations remain in current optical authentication studies of CR, AF, and related herbal materials. First, many studies focus on chemical profiling, metabolomics, or targeted quality markers, whereas compact Vis/NIR image-intensity spectra for rapid screening have received less systematic evaluation. Second, ROI-level records and sample-level decisions are often not clearly separated, which may inflate model performance when spectra from the same physical sample or acquisition group are split across training and testing sets. Third, most reported models rely on conventional preprocessing and direct classification, while fold-contained spectral augmentation for limited multi-band inputs is rarely examined. In addition, wavelength-level interpretation is frequently reported without retraining reduced-band models, making it difficult to judge whether selected wavelengths retain practical discriminative value. These gaps motivate the present study, which integrates grouped validation, sample-level reporting, lightweight diffusion-based spectral augmentation, and model-aligned wavelength evidence into a unified Vis/NIR authentication workflow.

The aim of this study was to evaluate whether compact Vis/NIR image-derived spectra can be used for reliable authentication of herbal materials under a leakage-controlled validation design. Two herbal-material datasets were considered: CR and visually similar substitute materials, and AF samples from four geographical origins. The study first compared common spectral preprocessing methods and representative classifiers. A lightweight diffusion module was then used as an auxiliary spectral augmentation method to examine whether class-related spectral variation could be better captured under limited sample conditions. Finally, informative LED bands were analyzed to explore whether a reduced number of spectral channels could still retain useful discrimination ability.

## 2. Materials and Methods

### 2.1. Data Sources and Analysis Levels

The plant materials used in this study included CR, three visually similar substitute materials, namely SGR, SR, and PR, and AF samples from four geographical origins. All materials were obtained as dried commercial herbal products from established suppliers or production enterprises. The CR-related materials were recorded as dried root fragments or slices, and the AF materials were recorded as dried fruit slices. Before spectral acquisition, the samples were visually inspected to remove severely damaged, moldy, or contaminated pieces. The spectral data were recorded from dried root fragments or slices rather than from intact plants. For each sample, the visible surface of the dried material was placed toward the imaging camera, and representative regions were selected for spectral extraction. The AF materials were dried fruit slices from four geographical origins.

**Figure 1:**
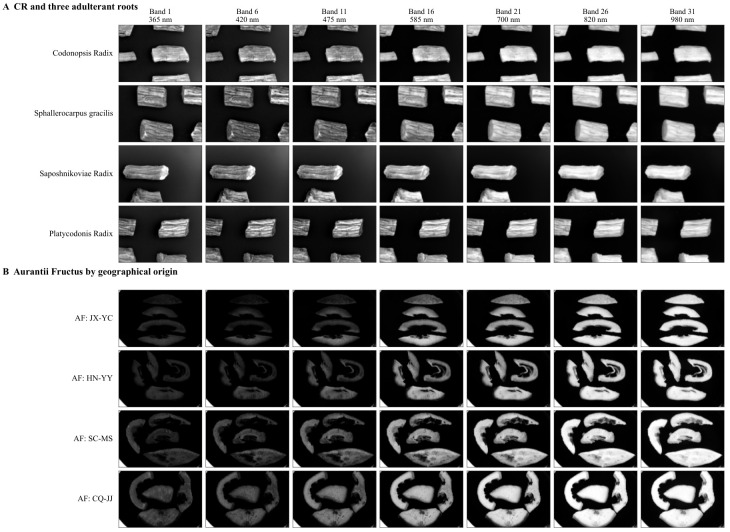
Representative multi-band images of the plant materials used in this study. (**A**) Codonopsis Radix (CR) and three visually similar root materials, including Sphallerocarpus gracilis (SGR), Saposhnikoviae Radix (SR), and Platycodonis Radix (PR). (**B**) Aurantii Fructus (AF) samples from four geographical origins, including Jiangxi–Yichun (JX-YC), Hunan–Yiyang (HN-YY), Sichuan–Meishan (SC-MS), and Chongqing–Jiangjin (CQ-JJ). The displayed columns correspond to representative LED bands, including Band 1 at 365 nm, Band 6 at 420 nm, Band 11 at 475 nm, Band 16 at 585 nm, Band 21 at 700 nm, Band 26 at 820 nm, and Band 31 at 980 nm. The CR-related data were recorded from dried root fragments or slices, whereas the AF data were recorded from dried fruit slices.

All materials were obtained as dried herbal products from established suppliers and production enterprises. All samples were collected from different batches to retain batch-level variation. The dried samples were placed in clean, dry containers and stored protected from light at room temperature, with a storage temperature of about 25 °C, relative humidity of 40–55%, and a storage duration of 3 days. No additional drying, heating, or chemical treatment was applied before imaging. Representative multi-band images of the plant materials are provided in Figure 1. The figure includes the four CR-related root materials and the four AF geographical-origin groups at selected LED bands. These images were added to clarify the physical form of the samples and to make the spectral acquisition procedure easier to understand.

The class labels of the CR dataset were assigned according to the declared material identity and were further checked by morphological inspection based on the characteristic appearance, color, texture, and tissue structure of each material. The AF geographical-origin labels were assigned according to the supplier-provided production-origin information and purchase records. Because the present study focused on Vis/NIR spectral screening rather than taxonomic revision or chemical quantification, the labels were treated as verified commercial-source labels for optical authentication analysis. The samples within each class were collected from independent commercial-source lots when available, so the dataset contained commercial-source variation rather than repeated measurements from a single homogeneous batch.

The CR dataset contained 600 region-of-interest (ROI)-derived mean gray-value spectral records from four classes, namely CR, SGR, SR, and PR. Each class included 150 ROI records. Each spectrum was constructed from 31 wavelength-specific LED images acquired at predefined nominal central wavelengths in the Vis/NIR region. The AF dataset contained 400 mean gray-value spectra from four geographical origins, with 100 spectra per origin and the same 31-band Vis/NIR range. AF origin labels were assigned according to the predefined class-coding scheme, and source-image group identifiers were retained for grouped validation.

Two analytical levels were used in this study. At the ROI level, each extracted region-of-interest mean gray-value spectrum was treated as one analytical record. At the sample level, CR ROI spectra sharing the same group identifier were averaged wavelength by wavelength to generate one sample-level spectrum, resulting in 52 CR sample-level spectra. For AF, each spectral record corresponded to an individual physical sample. Therefore, the 400 AF spectra were retained as 400 sample-level spectra, with 100 samples per origin. Because each acquisition scene contained four physical samples, source-image identifiers were used only to define grouped cross-validation splits and to prevent samples from the same image group from appearing in both training and testing folds.

### 2.2. Vis/NIR Image Acquisition System

The Vis/NIR spectral images were acquired using a self-built 31-band Vis/NIR imaging system equipped with wavelength-specific LED illumination, a CCD imaging unit, and a stationary sample platform. The CCD camera acquired grayscale images with a resolution of 1600×1200 pixels and 12-bit intensity depth. During image acquisition, the samples were placed on a fixed horizontal platform, and the relative position among the camera, LED illumination unit, and sample platform was kept unchanged to reduce position-related variation.

The imaging system used 31 discrete LED bands covering the visible and near-infrared regions. The central wavelengths of the 31 LED bands were 365, 380, 395, 400, 410, 420, 430, 440, 455, 465, 475, 500, 520, 535, 570, 585, 595, 620, 655, 680, 700, 730, 750, 770, 805, 820, 850, 880, 900, 940, and 980 nm. For each sample or ROI, one grayscale image was acquired at each LED band, and the 31 wavelength-specific images were used to construct a compact Vis/NIR mean gray-value spectrum.

The LED channels had finite emission bandwidths rather than monochromatic outputs, and the wavelength bandwidth of each channel was described by its nominal full width at half maximum (FWHM). The nominal FWHM values were 10, 10, 12, 12, 14, 15, 16, 18, 20, 22, 24, 30, 32, 35, 25, 18, 18, 20, 22, 25, 28, 30, 32, 35, 38, 38, 42, 45, 45, 50, and 55 nm for the 365, 380, 395, 400, 410, 420, 430, 440, 455, 465, 475, 500, 520, 535, 570, 585, 595, 620, 655, 680, 700, 730, 750, 770, 805, 820, 850, 880, 900, 940, and 980 nm channels, respectively. Exposure control was performed separately for each LED wavelength during white-reference calibration. The camera integration time was automatically adjusted using the white reference panel until the mean gray value of the calibration region reached approximately 70% of the maximum pixel intensity. After the wavelength-specific integration time had been determined, the same exposure setting was used for the corresponding dark-reference, white-reference, and sample images. The camera gain was kept constant throughout image acquisition, and no additional exposure adjustment was applied during sample imaging.

Before sample imaging, dark-reference and white-reference images were collected under the same acquisition geometry. The dark reference was obtained with the light path blocked, and the white reference was obtained using a standard white reference panel. Image correction was performed as follows:

(1) $$I_{c}=\frac{I_{s}-I_{d}}{I_{w}-I_{d}},$$

where $I_{c}$ is the corrected image intensity, $I_{s}$ is the raw sample image, $I_{d}$ is the dark-reference image, and $I_{w}$ is the white-reference image. This correction was used to reduce dark-current and illumination-related variation before extracting spectral features.

ROI extraction was performed on the corrected wavelength-specific images. The background was first removed according to the intensity difference between the sample and the background. The extracted ROI was then visually checked to exclude obvious background pixels, shadows, damaged regions, and edge artifacts. For each accepted ROI, the mean gray value was calculated at each of the 31 LED bands, resulting in a 31-dimensional ROI-derived mean gray-value spectrum. These spectra were used for subsequent preprocessing, grouped validation, classification, lightweight diffusion augmentation, and wavelength-evidence analysis.

### 2.3. Spectral Preprocessing

Four preprocessing strategies were evaluated: raw spectra, Savitzky–Golay smoothing, multiplicative scatter correction, and standard normal variate correction. Savitzky–Golay smoothing used a window length of 11, a third-order polynomial, and zero-order smoothing. Multiplicative scatter correction fitted the correction reference within the training fold only, and standard normal variate correction was applied spectrum by spectrum. Recent Vis/NIR and hyperspectral studies show that preprocessing choice and spectral representation can materially affect quality assessment and authentication performance when models are built from non-destructively acquired optical data [26,27,28].

**Figure 2:**
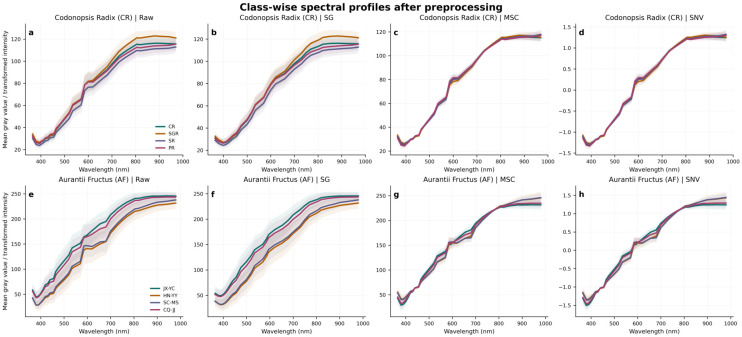
Class-wise spectral profiles after different preprocessing strategies. (**a**) CR-related materials with raw spectra; (**b**) CR-related materials after Savitzky–Golay (SG) smoothing; (**c**) CR-related materials after multiplicative scatter correction (MSC); (**d**) CR-related materials after standard normal variate (SNV) correction; (**e**) AF samples with raw spectra; (**f**) AF samples after SG smoothing; (**g**) AF samples after MSC; (**h**) AF samples after SNV correction. Shaded bands indicate one standard deviation within each class. CR = Codonopsis Radix, SGR = Sphallerocarpus gracilis, SR = Saposhnikoviae Radix, PR = Platycodonis Radix, and AF = Aurantii Fructus. AF origin abbreviations are JX-YC = Jiangxi–Yichun, HN-YY = Hunan–Yiyang, SC-MS = Sichuan–Meishan, and CQ-JJ = Chongqing–Jiangjin.

For visualization of spectral shape only, class-wise mean curves were plotted after applying raw, SG, MSC, and SNV preprocessing to the extracted spectra. These descriptive plots were not used as independent model evidence. The resulting class-wise spectral profiles are shown in Figure 2. In all classification experiments, preprocessing transformations were fitted within the training folds as described below.

### 2.4. Classification Design

Classifier selection used grouped cross-validation at the sample level. The classifier comparison included PCA-based SVM, XGBoost, KNN, and PLS-DA models, and direct-spectrum MLP, LSTM, 1D-CNN, ResNet1D, and Inception-ResNet1D models. Recent traditional Chinese medicine (TCM) and medicinal-material studies have combined near-infrared spectra, high-level data fusion, hyperspectral imaging, and machine learning for quality control and authentication tasks [29,30,31]. Because compact 31-band spectra can also be well suited to direct low-capacity classifiers, full-spectrum linear SVM variants were included as an expanded margin-based comparison and were carried forward to the main sample-level performance report when they achieved the highest grouped-validation macro-F1. Recent food-authentication studies using oil, wheat flour, and chicken samples show that both classical and flexible machine-learning models remain relevant for spectral classification [32,33,34]. Wavelength selection and reduced-band modeling were included because recent NIR studies show that carefully chosen spectral variables can improve sensor practicality while retaining discriminative information [35,36,37].

### 2.5. Lightweight Diffusion (LD) Spectral Augmentation Module

The LD module was used as an auxiliary spectral augmentation method for compact 31-band spectra. Its purpose was not to generate new botanical samples or to replace chemical measurements. Instead, it was used to learn small class-conditioned spectral variations from the training data and to generate denoised near-neighbor spectra within each training fold. In this way, the downstream classifier could be trained with slightly enriched spectral patterns while avoiding the use of test-fold information.

To strengthen the compact-spectrum classifier without introducing a large image-level network, a lightweight diffusion (LD) class-conditional one-dimensional denoising module was evaluated on the sample-level spectra. For a given outer training fold, let $x_{0,i}\in R^{d}$ denote the preprocessed and standardized spectrum of sample *i*, where d=31, and let $y_{i}\in \left\{1,\ldots ,C\right\}$ denote its class label. The LD module was designed as a small conditional denoising network rather than a high-capacity generative model. It used a two-hidden-layer multilayer perceptron with diffusion time-step embeddings and class embeddings. The number of diffusion steps was set to T=20.

The forward noising process followed a standard variance-preserving diffusion form. The noise schedule was linear:

(2) $$\beta_{t}=\beta_{\min}+\frac{t-1}{T-1}\left(\beta_{\max}-\beta_{\min}\right),\;t=1,\ldots ,T,$$

where $\beta_{\min}=1.0\times 10^{-4}$ and $\beta_{\max}=0.06$. With

(3) $$\alpha_{t}=1-\beta_{t},\;{\bar{\alpha }}_{t}=\prod_{s=1}^{t}\alpha_{s},$$

a noisy spectrum at step *t* was generated as

(4) $$x_{t}=\sqrt{{\bar{\alpha }}_{t}}x_{0}+\sqrt{1-{\bar{\alpha }}_{t}}\epsilon ,\;\epsilon ∼N\left(0,I_{d}\right).$$

The conditional denoising network $\epsilon_{\theta }\left(x_{t},t,c\right)$ was trained to predict the injected Gaussian noise from the noisy spectrum, the diffusion step, and the class label. For each training fold, the objective was

(5) $$L_{\mathrm{LD}}\left(\theta \right)=E_{\left(x_{0},y\right)\in D_{\mathrm{tr}},\;t,\;\epsilon }\left[\frac{1}{d}{∥\epsilon -\epsilon_{\theta }\left(x_{t},t,y\right)∥}_{2}^{2}\right].$$

This formulation was suitable for compact spectra because the network learned local class-conditioned spectral correction patterns in a 31-dimensional space, rather than attempting to model full image-level spatial distributions.

The LD module was fitted separately inside each outer training fold after spectral preprocessing, feature normalization, and standard scaling had been fitted on that training fold. LD augmentation was implemented in two forms. In synthetic spectral augmentation, noisy versions of training spectra were denoised by the learned LD module to form near-neighbor synthetic spectra. Given a noisy training spectrum $x_{t,i}$ generated from $x_{0,i}$, the denoised spectral estimate was calculated as

(6) $${\hat{x}}_{0,i}=\frac{x_{t,i}-\sqrt{1-{\bar{\alpha }}_{t}}\;\epsilon_{\theta }\left(x_{t,i},t,y_{i}\right)}{\sqrt{{\bar{\alpha }}_{t}}}.$$

Only denoised spectra generated from training-fold samples were appended to the classifier training set:

(7) $$D_{\mathrm{aug}}=D_{\mathrm{tr}}\cup \left\{\left({\hat{x}}_{0,i}^{\left(m\right)},y_{i}\right)|\left(x_{0,i},y_{i}\right)\in D_{\mathrm{tr}},\;m=1,\ldots ,M\right\}.$$

Thus, the synthetic spectra acted as class-preserving near-neighbor perturbations in the standardized spectral space.

In evidence-channel augmentation, the trained LD module was used as a frozen class-conditional spectral evidence extractor. For a spectrum $x_{0}$ and a candidate class *c*, the class-wise denoising residual was computed by testing the same spectrum under each candidate label:

(8) $$r_{c}\left(x_{0}\right)=\frac{1}{M}\sum_{m=1}^{M}\frac{1}{d}{∥\epsilon^{\left(m\right)}-\epsilon_{\theta }\left(x_{t}^{\left(m\right)},t^{\left(m\right)},c\right)∥}_{2}^{2},$$

where

(9) $$x_{t}^{\left(m\right)}=\sqrt{{\bar{\alpha }}_{t^{\left(m\right)}}}x_{0}+\sqrt{1-{\bar{\alpha }}_{t^{\left(m\right)}}}\epsilon^{\left(m\right)}.$$

The residual was converted into a numerical evidence score:

(10) $$e_{c}\left(x_{0}\right)=-\log\left(r_{c}\left(x_{0}\right)+\delta \right),$$

where δ is a small positive constant for numerical stability. A lower denoising residual indicates that the spectrum is more compatible with the tested class condition, and therefore gives a larger evidence score. The final evidence-augmented input vector was

(11) $$z={\left[x_{0}^{⊤},e_{1}\left(x_{0}\right),e_{2}\left(x_{0}\right),\ldots ,e_{C}\left(x_{0}\right)\right]}^{⊤}.$$

The vector z was then used as the input to the downstream classifier.

In both LD forms, test-fold spectra were never used to train the LD module or to generate synthetic training spectra. For the evidence-channel branch, test-fold evidence was calculated only by the frozen LD module trained on the corresponding training fold. The final LD setting for each dataset was selected by grouped-validation macro-F1 and compared with the corresponding non-LD branch. To assess LD behavior beyond the final selected classifier, all evaluated classifiers were also evaluated in a saved model-family LD re-evaluation, with baseline F1 values synchronized to the non-LD model table for consistency.

### 2.6. Validation and Metrics

For ROI-level analysis, stratified grouped cross-validation was used so that records sharing the same group identifier were kept within the same fold. For sample-level analysis, CR group-averaged samples and AF per-sample spectra were split under the same grouped cross-validation framework; for AF, the four samples from the same source image were kept in the same fold. All preprocessing transformations, PCA models, when used, LD modules, and classifier parameters were fitted only on the training portion of each fold and then applied to the corresponding test portion. Model performance was assessed using accuracy, macro precision, macro recall, and macro-F1.

For a class *c*, let $TP_{c}$, $FP_{c}$, and $FN_{c}$ denote true positives, false positives, and false negatives. With *C* classes and *N* test records, the reported metrics were calculated as follows:

(12) $$\begin{array}{cc} \mathrm{Accuracy} & =\frac{1}{N}\sum_{i=1}^{N}1\left(y_{i}={\hat{y}}_{i}\right), \end{array}$$

(13) $$\begin{array}{cc} {\mathrm{Precision}}_{macro} & =\frac{1}{C}\sum_{c=1}^{C}\frac{TP_{c}}{TP_{c}+FP_{c}}, \end{array}$$

(14) $$\begin{array}{cc} {\mathrm{Recall}}_{macro} & =\frac{1}{C}\sum_{c=1}^{C}\frac{TP_{c}}{TP_{c}+FN_{c}}, \end{array}$$

(15) $$\begin{array}{cc} F1_{macro} & =\frac{1}{C}\sum_{c=1}^{C}\frac{2\;{\mathrm{Precision}}_{c}\;{\mathrm{Recall}}_{c}}{{\mathrm{Precision}}_{c}+{\mathrm{Recall}}_{c}}. \end{array}$$

When a denominator was zero for a class, the corresponding class-level precision, recall, or F1 term was set to 0, matching the implementation used for the saved experiment outputs.

### 2.7. Wavelength-Level Interpretation

Wavelength-level interpretation was aligned with the model branches used in the reduced-band experiments. For the final Linear SVM, wavelengths were ranked according to the mean absolute standardized coefficients across outer training folds. This score reflects the contribution of each wavelength to the linear decision margin. For the MLP branch, wavelengths were ranked using held-out permutation importance. One wavelength variable was permuted at a time in the outer test fold, and the resulting decrease in macro-F1 was used as the importance score.

The top 5, top 8, and top 12 wavelengths were selected separately for each dataset and model branch. The same model family was then retrained using only the selected wavelengths under the original grouped-validation protocol. This design ensured that the reduced-band results were directly comparable with the corresponding full-spectrum models.

LD was used only as an auxiliary spectral augmentation and evidence-enhancement module. It was not used to rank individual wavelengths, so the wavelength evidence remained directly tied to the supervised classifiers. Grad-CAM was not used because the reported wavelength-ranking branches were Linear SVM and MLP, which do not provide the convolutional activation maps required for Grad-CAM analysis.

## 3. Results

### 3.1. ROI-Level and Sample-Level Data Sets

**Figure 3:**
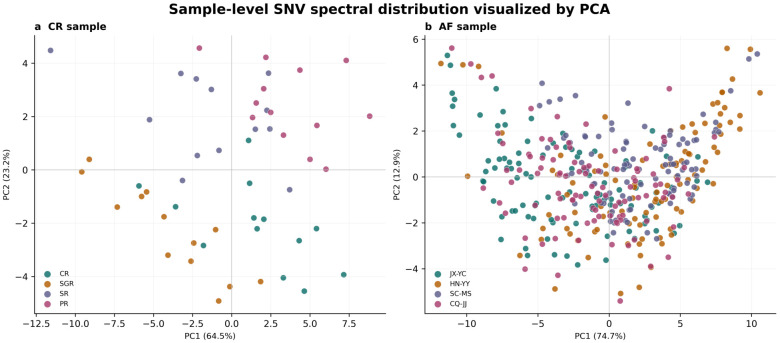
PCA score plots of SNV-preprocessed sample-level spectra. (**a**) PCA projection of the CR-related materials, including Codonopsis Radix (CR), Sphallerocarpus gracilis (SGR), Saposhnikoviae Radix (SR), and Platycodonis Radix (PR). (**b**) PCA projection of the AF samples from four geographical origins, including Jiangxi–Yichun (JX-YC), Hunan–Yiyang (HN-YY), Sichuan–Meishan (SC-MS), and Chongqing–Jiangjin (CQ-JJ).

The ROI-level and sample-level definitions are summarized in Table 1. CR sample-level analysis reduced the number of modeling records by averaging ROI records within each group. AF sample-level analysis retained all 400 sample spectra, with the source-image group used only to prevent grouped cross-validation leakage. PCA score plots of the SNV-preprocessed spectra were generated for the CR and AF datasets, as shown in Figure 3. The classification and wavelength-evidence results below are reported at the sample level.

### 3.2. Classification Performance

The classification results are presented from simple models to more flexible neural models. This order was used to show whether the compact 31-band spectra required complex modeling or could already be effectively analyzed by lower-capacity spectral classifiers. Because the inputs were mean gray-value spectra rather than full image cubes, the deep-learning models were evaluated as comparative baselines rather than assumed to be superior a priori.

For CR at the sample level, the non-LD grouped-validation grid selected Linear SVM; accuracy = 0.9231 and macro-F1 = 0.9238.

For AF at the sample level, the non-LD grouped-validation grid selected Linear SVM; accuracy = 0.9025 and macro-F1 = 0.9018.

The performance of the requested machine-learning and deep-learning models is reported first in Table 2.

**Figure 4:**
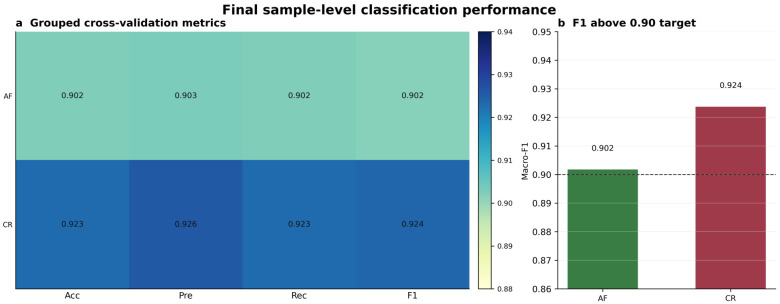
Selected non-LD sample-level grouped-validation performance of the final Linear SVM models. (**a**) Heatmap of grouped cross-validation metrics, including accuracy (Acc), macro precision (Pre), macro recall (Rec), and macro-F1 (F1), for the AF and CR datasets. (**b**) Macro-F1 values of the selected final models for AF and CR, with the dashed line indicating the 0.90 reference target.

Both sample-level tasks exceeded 0.90 accuracy and macro-F1 under grouped cross-validation before adding LD. The selected non-LD sample-level performance values are summarized in Table 3 and visualized in Figure 4.

After the primary non-LD evaluation, lightweight diffusion (LD) augmentation was evaluated across all model families to examine whether class-conditioned denoising could provide general auxiliary spectral information beyond a single selected pipeline. Table 4 summarizes the macro-F1 changes after LD augmentation for SVM, XGBoost, KNN, PLS-DA, MLP, 1D-CNN, LSTM, ResNet1D, and Inception-ResNet1D.

Compared with the corresponding baseline results in Table 2, the fold-contained LD module improved macro-F1 in all 18 model–dataset comparisons. For CR, the mean macro-F1 increased from 0.7346 to 0.8567, corresponding to an absolute gain of 0.1221 and a relative gain of 16.63%. For AF, the mean macro-F1 increased from 0.7258 to 0.7811, with an absolute gain of 0.0553 and a relative gain of 7.62%. Across the two datasets, the overall mean macro-F1 increased from 0.7302 to 0.8189, giving an average absolute improvement of 0.0887.

The gains were especially clear in the deep-learning branch. For CR, LD increased the macro-F1 of 1D-CNN from 0.4767 to 0.8071 and that of Inception-ResNet1D from 0.5214 to 0.8260. For AF, the largest improvement was also observed for 1D-CNN, with macro-F1 increasing from 0.5998 to 0.7046. These results indicate that LD-based spectral augmentation provided effective regularization for flexible neural models under limited independent training groups. Meanwhile, the SVM branch remained the strongest LD-augmented classifier for both datasets, reaching macro-F1 values of 0.9427 for CR and 0.9197 for AF. Therefore, LD acted as a general auxiliary spectral-evidence enhancement module rather than improving only one selected classifier.

The deep-learning branch was not uniformly superior under grouped validation. This result is plausible for the present data structure rather than a contradiction of the modeling design. The classifier inputs were compact 31-band mean gray-value spectra, not full hyperspectral image cubes; therefore, spatial morphology and local texture cues that could benefit convolutional or recurrent deep networks were not available to the deep models. CR also contained only 52 sample-level spectra after ROI-group averaging, and AF was evaluated with image-grouped folds so that the four samples from the same source image were not split across training and testing. Under these conditions, the number of independent training groups was limited, and low-capacity supervised spectral classifiers such as linear SVM and PLS-DA were better matched to the smooth low-dimensional spectral vectors. The strongest MLP result improved after refitting the training fold with the validation-selected epoch count, but the final sample-level classifier remained the linear SVM for both datasets.

To evaluate model stability and the meaningfulness of the observed performance differences, an additional statistical analysis was conducted under repeated grouped validation. Because the CR sample-level dataset was compact, uncertainty statistics were reported for all evaluated CR model families. For AF, the statistical comparison was focused on the selected final non-LD and LD-augmented SVM models. The grouped-validation procedure was repeated 30 times with different random seeds while preserving the group constraints. Repeated macro-F1 values, 95% confidence intervals, fold-wise macro-F1 ranges, class-wise F1-score ranges, and paired statistical comparisons were summarized in Table 5.

As shown in Table 5, the CR uncertainty analysis showed that Linear SVM and PLS-DA maintained stable performance under repeated grouped validation, whereas higher-capacity deep-learning models showed larger variation and lower mean macro-F1 values under the compact 31-channel sample-level setting. After LD augmentation, the SVM macro-F1 increased from 0.9235±0.0268 to 0.9419±0.0217 for CR and from 0.9016±0.0242 to 0.9193±0.0205 for AF. The paired statistical comparisons indicated that the LD-augmented SVM provided meaningful improvements over the corresponding non-LD Linear SVM. These results provide fold-wise, repeated-validation, confidence-interval, class-wise, and statistical evidence for interpreting the reported model differences.

**Figure 5:**
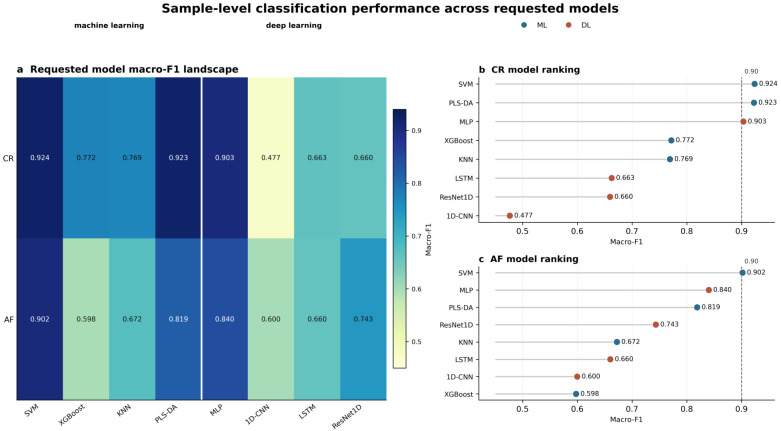
Sample-level performance landscape across the requested machine-learning and deep-learning models. (**a**) summarizes macro-F1 across models and datasets; (**b**,**c**) rank models within CR and AF, respectively. The dashed vertical line marks the 0.90 macro-F1 reference.

**Figure 6:**
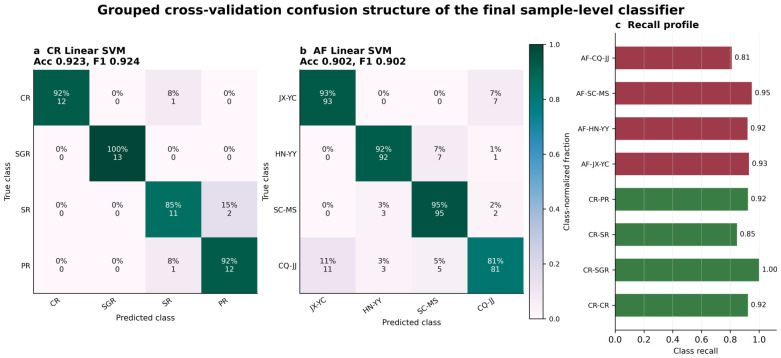
Grouped cross-validation confusion structure of the final sample-level Linear SVM classifiers. (**a**) Row-normalized confusion matrix for the CR-related material classification task, with each cell annotated by percentage and sample count. (**b**) Row-normalized confusion matrix for the AF geographical-origin classification task, with each cell annotated by percentage and sample count. (**c**) Class-wise recall profile for the CR-related classes and AF origin classes; green bars indicate CR-related classes and red bars indicate AF origin classes.

The model-level performance landscape in Figure 5 visualizes the same sample-level model comparison as Table 2, while Figure 6 shows the class-level error structure of the final Linear SVM models.

### 3.3. Wavelength Evidence and Top-Band Subset Tests

**Figure 7:**
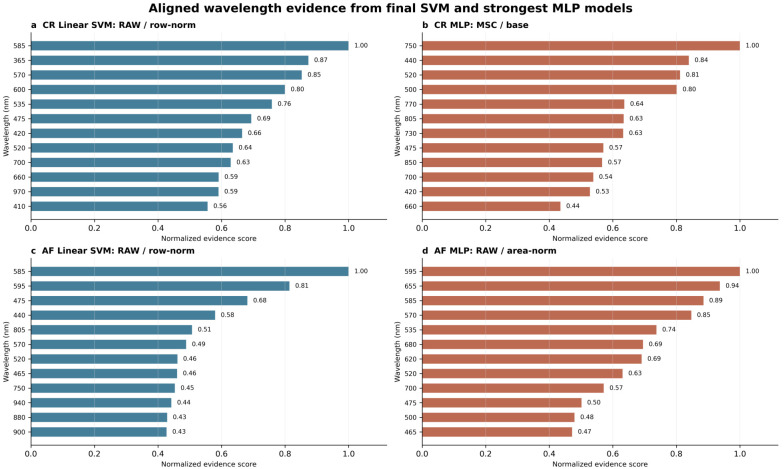
Aligned sample-level wavelength evidence for the final Linear SVM and strongest MLP models. (**a**) Top wavelength evidence for the CR Linear SVM branch under the RAW/row-normalized setting, based on normalized mean absolute standardized coefficients. (**b**) Top wavelength evidence for the CR MLP branch under the MSC/base setting, based on normalized held-out permutation macro-F1 decrease. (**c**) Top wavelength evidence for the AF Linear SVM branch under the RAW/row-normalized setting, based on normalized mean absolute standardized coefficients. (**d**) Top wavelength evidence for the AF MLP branch under the RAW/area-normalized setting, based on normalized held-out permutation macro-F1 decrease.

Wavelength evidence was analyzed to identify which LED bands contributed most to the spectral discrimination. The purpose of this analysis was to examine whether certain visible or near-infrared regions carried stronger classification information under the present imaging system. The normalized wavelength-evidence scores for the final Linear SVM and strongest MLP branches are visualized in Figure 7.

The sample-level wavelength evidence is summarized in Table 6. To keep the finite-band analysis consistent with the model-performance tables, wavelength ranking was reported for the final Linear SVM and for the strongest MLP branch.

The top-wavelength subset tests are reported in Table 7. CR Linear SVM top-five, top-eight, and top-12 macro-F1 values were 0.7415, 0.9615, 0.9221, respectively; CR MLP top-five, top-eight, and top-12 macro-F1 values were 0.5069, 0.5963, 0.7054, respectively; AF Linear SVM top-five, top-eight, and top-12 macro-F1 values were 0.7092, 0.7800, 0.8392, respectively; AF MLP top-five, top-eight, and top-12 macro-F1 values were 0.8003, 0.8224, 0.8475, respectively.

**Figure 8:**
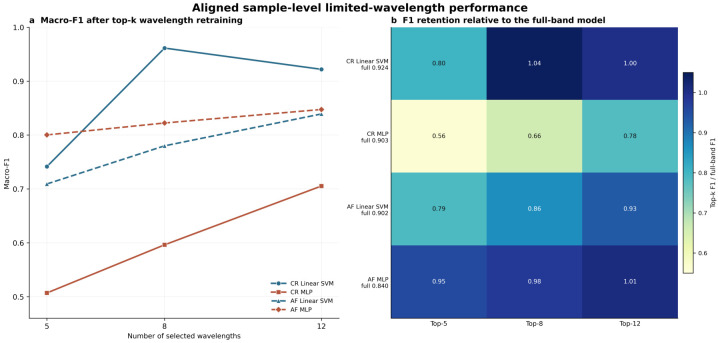
Aligned sample-level limited-wavelength performance for Linear SVM and MLP models after retraining with the top-5, top-8, and top-12 wavelength subsets. (**a**) Macro-F1 values obtained after retraining the CR Linear SVM, CR MLP, AF Linear SVM, and AF MLP branches with different numbers of selected wavelengths. (**b**) F1 retention heatmap relative to the corresponding full-band model, calculated as the top-k macro-F1 divided by the full-band macro-F1.

The limited-wavelength retraining results are further visualized in Figure 8, which compares the absolute macro-F1 values and the relative retention of full-band performance across the top-5, top-8, and top-12 wavelength subsets. The wavelength-ranking results should be interpreted as classifier-based evidence for informative LED bands and spectral regions, rather than as assignments of isolated single bands to specific compounds. Unlike mid-infrared or NMR spectra, UV/Vis/NIR responses are broad, overlapping, and influenced by combined chemical and physical factors. Therefore, the reduced-band tests were not designed to evaluate one single wavelength alone, but to examine whether subsets of multiple selected LED bands could retain useful discriminative information. The selected top-five, top-eight, and top-12 subsets were therefore interpreted as compact spectral-region combinations within the present 31-band LED imaging system.

To examine whether the LD-related gains and reduced-band results were affected by arbitrary perturbation or wavelength-selection bias, a focused diagnostic ablation analysis was added for the final SVM branch. For LD augmentation, the non-LD Linear SVM was compared with Gaussian-noise augmentation, the final LD-augmented SVM, and a shuffled-label LD control. For wavelength selection, the selected top-12 LED-band subset was compared with the full 31-band baseline, random 12-band subsets, and low-importance 12-band subsets under the same grouped-validation framework. The results are summarized in Table 8.

As shown in Table 8, Gaussian-noise augmentation produced only minor gains over the non-LD baseline, whereas the final LD-augmented SVM achieved higher macro-F1 values on both datasets. The shuffled-label LD control did not improve performance, suggesting that the LD gain was related to class-conditioned spectral information rather than arbitrary perturbation. For wavelength selection, the selected top-12 LED-band subsets retained competitive performance relative to the full 31-band input and performed better than the random-band and low-importance controls. These results indicate that the wavelength-ranking procedure preserved useful discriminative information, while the reduced-band findings should still be interpreted as within-system evidence under the present 31-band LED imaging configuration rather than as proof of finalized reduced-band sensor design.

### 3.4. Internal and External Validation

To evaluate model quality beyond grouped cross-validation, a group-aware 70/30 internal train/test validation and an independent external validation were added. In the 70/30 validation, 70% of the original sample-level data were used for training and 30% for testing, while preserving class balance and group constraints to avoid information leakage. This procedure was repeated 30 times with different random seeds, and the results were summarized as mean ± standard deviation. For external validation, newly collected independent samples were used, including 160 CR-related samples with 40 samples per class and 200 AF samples with 50 samples per geographical origin. These external samples were not used for model training, parameter selection, LD fitting, wavelength selection, or model optimization.

As shown in Table 9, the validation results were consistent with the grouped cross-validation trends. In the repeated 70/30 internal validation, the Linear SVM achieved macro-F1 values of 0.9176±0.0430 for CR and 0.8948±0.0235 for AF, while the LD-augmented SVM achieved 0.9364±0.0372 and 0.9141±0.0198, respectively. In the independent external validation, the LD-augmented SVM achieved macro-F1 values of 0.9247 for CR and 0.9195 for AF. These results provide additional train/test and independent-sample evidence for the stability of the selected model.

### 3.5. Tentative Spectral-Chemical Interpretation of Informative LED Bands

The informative LED bands were further interpreted from a spectral-chemical perspective. Because the present data were ROI-derived mean gray-value spectra rather than calibrated absorbance spectra or quantitative chemical measurements, the selected bands were not assigned to single compounds. Instead, they were grouped into broader visible and near-infrared regions and tentatively associated with major optical, chemical, and physicochemical factors that may contribute to the discrimination of CR-related materials and AF origins. As summarized in Table 10, visible bands were mainly related to color, browning, pigments, and phenolic/flavonoid-associated optical responses, whereas near-infrared bands were more likely associated with moisture-related O-H responses, broad C-H/O-H/N-H overtone or combination information, and matrix scattering. These interpretations provide chemical and physicochemical context for the wavelength-evidence results while avoiding overstatement beyond the measurement type and spectral resolution of the present imaging system.

## 4. Discussion

The results show that compact Vis/NIR mean gray-value spectra can provide useful discrimination information for the studied herbal materials. From an analytical perspective, the main finding is that relatively simple spectral classifiers, especially Linear SVM and PLS-DA, were well suited to the 31-band image-derived spectra. This is reasonable because the present inputs contained spectral intensity information but did not include spatial texture or full hyperspectral image information. Therefore, the benefit of high-capacity neural models was limited under the present sample size and data structure.

The current sample-level results support the feasibility of using compact Vis/NIR mean gray-value spectra for classification in the two herbal medicine datasets. Under grouped validation, the selected CR and AF configurations both exceeded 0.90 accuracy and macro-F1. These statements are limited to the present datasets, preprocessing choices, split strategy, and model settings; they should not be generalized beyond the validated experimental setting. Recent non-destructive authentication studies on cachaca, saffron, and peanut adulteration similarly show that the success of spectral screening depends on the material matrix, validation design, and analytical target [38,39,40].

The LD experiment added a fold-contained regularization layer to the compact spectral workflow. Across the evaluated model families, LD increased the overall mean macro-F1 from 0.7302 to 0.8189. The improvement was especially large for the neural classifiers, which are more sensitive to limited independent training groups. This pattern suggests that class-conditioned denoising supplied local spectral perturbations and denoising-error evidence that stabilized flexible classifiers in the 31-band input space. These results should be interpreted as spectral evidence of regularization rather than as proof that a large generative model learned the full distribution of herbal materials.

The observation that several deep-learning models underperformed the best machine-learning models should be interpreted in relation to the experimental input representation and validation design. Deep learning is often advantageous when the model can learn from many independent training examples or from rich spatial-spectral image structure. In this study, however, each record was a 31-dimensional mean spectrum. The averaging step reduced noise and made the spectra suitable for compact classifiers, but it also removed within-sample spatial patterns that 1D-CNN, ResNet1D, Inception-ResNet1D, and LSTM models might otherwise exploit. The CR sample-level set was especially small, with 13 spectra per class after group averaging, making high-capacity networks more vulnerable to fold-to-fold variance and overfitting. For AF, the 400 spectra came from 100 source-image groups, and grouped cross-validation deliberately prevented samples from the same image group from appearing in both training and testing. This group-aware evaluation is stricter than a random row-level split and reduces the apparent advantage of flexible models. Linear SVM and PLS-DA are therefore competitive here because they impose simpler supervised structures on smooth low-dimensional spectra, which can improve generalization when independent sample groups are limited. Recent reviews of intelligent sensory systems for TCM quality evaluation also emphasize that model choice should match the available signal, sample size, and inspection scenario [41].

The aligned top-wavelength experiments show that reduced spectral inputs can retain part of the full-spectrum performance, but the pattern differs by model and dataset. For CR, the Linear SVM top-8 subset exceeded the full-band grouped-validation macro-F1 in this run, whereas the MLP subsets lost substantial performance as the input was narrowed. For AF, the Linear SVM top-12 subset retained most but not all of the full-spectrum F1, while the MLP top-12 subset slightly exceeded its saved full-band MLP result. These results should be interpreted as model evidence rather than direct chemical assignment; chemical explanations require verified chemical references or complementary measurements.

Several limitations should also be noted. The AF geographical-origin labels reflected the declared commercial origins, and broader multi-year, multi-batch, and independently certified samples are still required to further evaluate origin-level robustness. The present spectral data were mean image-intensity spectra rather than calibrated quantitative reflectance or chemical measurements. Therefore, the reported classification results should be interpreted as evidence for Vis/NIR optical screening under the present sample-source and validation conditions, not as direct chemical or taxonomic verification. In addition, the spectral inputs were ROI-derived mean gray-value spectra rather than fully calibrated reflectance spectra. Therefore, the reduced-band results should be interpreted as preliminary within-system evidence of discriminative LED-band information, not as direct proof of cross-instrument transferability or finalized reduced-band sensor design.

## 5. Conclusions

This study established an interpretable Vis/NIR spectral learning framework for the non-destructive authentication of CR and AF. The framework combined compact 31-band image-intensity spectra, fold-contained preprocessing, grouped cross-validation, LD-based spectral augmentation, and model-aligned wavelength interpretation. Under sample-level grouped validation, the strongest non-LD Linear SVM models achieved macro-F1 values of 0.9238 for CR and 0.9018 for AF. After LD augmentation, the best LD-augmented SVM models further increased the macro-F1 values to 0.9427 and 0.9197, respectively. Across all evaluated model–dataset combinations, LD increased the overall mean macro-F1 from 0.7302 to 0.8189, indicating that class-conditioned denoising can provide effective auxiliary spectral regularization for compact Vis/NIR inputs.

The wavelength-level analysis further showed that discriminative information was concentrated in selected Vis/NIR regions. Reduced-band retraining retained substantial classification performance, and some reduced-band settings matched or exceeded the corresponding full-band models. These results indicate that compact Vis/NIR image-intensity spectra can support accurate, interpretable, and leakage-controlled authentication of herbal materials. The proposed workflow also provides a practical basis for reduced-band sensor design and lightweight optical screening.

## Figures and Tables

**Table 1:** ROI-level and sample-level definitions used for modeling.

Dataset	ROI Records	Sample-Level Spectra	Bands	Sample-Level Class Counts	Label Evidence
CR	600	52	31	CR = 13; SGR = 13; SR = 13; PR = 13	Final study class map
AF	400	400	31	Jiangxi–Yichun = 100; Hunan–Yiyang = 100; Sichuan–Meishan = 100; Chongqing–Jiangjin = 100	Predefined origin labels; source-image groups retained for grouped validation

**Table 2:** Sample-level performance of the evaluated machine-learning and deep-learning models.

Dataset	Branch	Model	Acc	Pre	Rec	F1
CR	ML	SVM	0.9231	0.9258	0.9231	0.9238
CR	ML	XGBoost	0.7692	0.7772	0.7692	0.7716
CR	ML	KNN	0.7692	0.7692	0.7692	0.7692
CR	ML	PLS-DA	0.9231	0.9273	0.9231	0.9226
CR	DL	MLP	0.9038	0.9094	0.9038	0.9034
CR	DL	1D-CNN	0.5385	0.4317	0.5385	0.4767
CR	DL	LSTM	0.6923	0.6868	0.6923	0.6627
CR	DL	ResNet1D	0.6538	0.6843	0.6538	0.6597
CR	DL	Inception-ResNet1D	0.5385	0.5461	0.5385	0.5214
AF	ML	SVM	0.9025	0.9027	0.9025	0.9018
AF	ML	XGBoost	0.6000	0.5965	0.6000	0.5978
AF	ML	KNN	0.6750	0.6725	0.6750	0.6724
AF	ML	PLS-DA	0.8225	0.8234	0.8225	0.8188
AF	DL	MLP	0.8400	0.8415	0.8400	0.8401
AF	DL	1D-CNN	0.6000	0.6019	0.6000	0.5998
AF	DL	LSTM	0.6650	0.6606	0.6650	0.6603
AF	DL	ResNet1D	0.7450	0.7475	0.7450	0.7434
AF	DL	Inception-ResNet1D	0.6975	0.6984	0.6975	0.6977

**Table 3:** Selected non-LD sample-level classification performance under grouped cross-validation.

Dataset	Model	Acc	Pre	Rec	F1
CR	Linear SVM	0.9231	0.9258	0.9231	0.9238
AF	Linear SVM	0.9025	0.9027	0.9025	0.9018

**Table 4:** Macro-F1 improvement of sample-level machine-learning and deep-learning models after fold-contained lightweight diffusion (LD) augmentation.

Dataset	Branch	Model	Baseline F1	LD F1	ΔF1	Relative Gain (%)
CR	ML	SVM	0.9238	**0.9427**	+0.0189	+2.05
CR	ML	XGBoost	0.7716	0.8278	+0.0562	+7.28
CR	ML	KNN	0.7692	0.8085	+0.0393	+5.11
CR	ML	PLS-DA	0.9226	0.9420	+0.0194	+2.10
CR	DL	MLP	0.9034	0.9234	+0.0200	+2.21
CR	DL	1D-CNN	0.4767	0.8071	**+0.3304**	**+69.31**
CR	DL	LSTM	0.6627	0.7859	+0.1232	+18.59
CR	DL	ResNet1D	0.6597	0.8468	+0.1871	+28.36
CR	DL	Inception-ResNet1D	0.5214	0.8260	+0.3046	+58.42
CR mean			0.7346	0.8567	+0.1221	+16.63
AF	ML	SVM	0.9018	**0.9197**	+0.0179	+1.98
AF	ML	XGBoost	0.5978	0.6721	+0.0743	+12.43
AF	ML	KNN	0.6724	0.7142	+0.0418	+6.22
AF	ML	PLS-DA	0.8188	0.8432	+0.0244	+2.98
AF	DL	MLP	0.8401	0.8728	+0.0327	+3.89
AF	DL	1D-CNN	0.5998	0.7046	**+0.1048**	**+17.47**
AF	DL	LSTM	0.6603	0.7168	+0.0565	+8.56
AF	DL	ResNet1D	0.7434	0.8017	+0.0583	+7.84
AF	DL	Inception-ResNet1D	0.6977	0.7846	+0.0869	+12.46
AF mean			0.7258	0.7811	+0.0553	+7.62
Overall mean			0.7302	0.8189	+0.0887	+12.15

Baseline F1 values are taken from Table 2. ΔF1 is calculated as LD F1 minus baseline F1. Relative gain is calculated as ΔF1 divided by baseline F1. Bold LD F1 values indicate the best LD-augmented classifier within each dataset, and bold gain values indicate the largest improvement within each dataset.

**Table 5:** Uncertainty and statistical analysis under repeated grouped validation.

Dataset	Model	Repeated Macro-F1	95% CI	F1 Range	*p* Value
CR	Linear SVM	0.9235±0.0268	0.9135–0.9335	Fold: 0.9091–0.9444; Class: 0.884–0.963	–
CR	PLS-DA	0.9211±0.0283	0.9105–0.9317	Fold: 0.8615–0.9667; Class: 0.882–0.960	0.781
CR	MLP	0.9019±0.0351	0.8888–0.9150	Fold: 0.8231–0.9538; Class: 0.858–0.948	0.047
CR	XGBoost	0.7702±0.0556	0.7494–0.7910	Fold: 0.6538–0.8615; Class: 0.704–0.836	<0.001
CR	KNN	0.7672±0.0508	0.7482–0.7862	Fold: 0.6615–0.8462; Class: 0.710–0.829	<0.001
CR	LSTM	0.6615±0.0714	0.6348–0.6882	Fold: 0.5154–0.8000; Class: 0.542–0.769	<0.001
CR	ResNet1D	0.6578±0.0761	0.6294–0.6862	Fold: 0.4923–0.7923; Class: 0.548–0.756	<0.001
CR	1D-CNN	0.4759±0.0876	0.4432–0.5086	Fold: 0.3077–0.6308; Class: 0.310–0.615	<0.001
CR	Inception-ResNet1D	0.5208±0.0893	0.4874–0.5542	Fold: 0.3385–0.6846; Class: 0.382–0.658	<0.001
CR	LD-augmented SVM	0.9419±0.0217	0.9338–0.9500	Fold: 0.9286–0.9545; Class: 0.916–0.975	0.018
AF	Linear SVM	0.9016±0.0242	0.8929–0.9103	Fold: 0.8839–0.9250; Class: 0.873–0.931	–
AF	LD-augmented SVM	0.9193±0.0205	0.9120–0.9266	Fold: 0.9089–0.9375; Class: 0.896–0.944	0.024

**Table 6:** Aligned sample-level top wavelength regions identified for the final SVM and strongest MLP models.

Level	Dataset	Model	Evidence Score	Top Wavelengths (nm)
sample	CR	Linear SVM	absolute standardized coefficient	585; 365; 570; 600; 535; 475; 420; 520; 700; 660; 980; 410
sample	CR	MLP	held-out permutation F1 decrease	750; 440; 520; 500; 770; 805; 730; 475; 850; 700; 420; 660
sample	AF	Linear SVM	absolute standardized coefficient	585; 595; 475; 440; 805; 570; 520; 465; 750; 940; 880; 900
sample	AF	MLP	held-out permutation F1 decrease	595; 655; 585; 570; 535; 680; 620; 520; 700; 475; 500; 465

**Table 7:** Reduced-band sample-level performance after retraining aligned SVM and MLP models with the top-5, top-8, and top-12 wavelength subsets.

Dataset	Model	Top-*k*	Selected Wavelengths (nm)	Acc	Full-Band F1	Top-*k* F1	Retention (%)
CR	Linear SVM	5	585; 365; 570; 600; 535	0.7500	0.9238	0.7415	80.27
CR	Linear SVM	8	585; 365; 570; 600; 535; 475; 420; 520	0.9615	0.9238	**0.9615**	**104.08**
CR	Linear SVM	12	585; 365; 570; 600; 535; 475; 420; 520; 700; 660; 980; 410	0.9231	0.9238	0.9221	99.82
CR	MLP	5	750; 440; 520; 500; 770	0.5192	0.9034	0.5069	56.11
CR	MLP	8	750; 440; 520; 500; 770; 805; 730; 475	0.5962	0.9034	0.5963	66.01
CR	MLP	12	750; 440; 520; 500; 770; 805; 730; 475; 850; 700; 420; 660	0.7115	0.9034	0.7054	78.08
AF	Linear SVM	5	585; 595; 475; 440; 805	0.7200	0.9018	0.7092	78.64
AF	Linear SVM	8	585; 595; 475; 440; 805; 570; 520; 465	0.7800	0.9018	0.7800	86.49
AF	Linear SVM	12	585; 595; 475; 440; 805; 570; 520; 465; 750; 940; 880; 900	0.8400	0.9018	0.8392	93.06
AF	MLP	5	595; 655; 585; 570; 535	0.8000	0.8401	0.8003	95.26
AF	MLP	8	595; 655; 585; 570; 535; 680; 620; 520	0.8225	0.8401	0.8224	97.89
AF	MLP	12	595; 655; 585; 570; 535; 680; 620; 520; 700; 475; 500; 465	0.8475	0.8401	**0.8475**	**100.88**

Full-band F1 values are taken from the corresponding full-spectrum models in Table 2. Retention is calculated as top-*k* F1 divided by full-band F1. Bold values indicate reduced-band settings that matched or exceeded the corresponding full-band model.

**Table 8:** Focused diagnostic ablation analysis of LD augmentation and wavelength selection for the final SVM branch.

Dataset	Analysis	Baseline/Simple Control	Final/Selected Setting	Negative or Random Control
CR	LD	Non-LD: 0.9238; Gaussian noise: 0.9276	Final LD: 0.9427	Shuffled-label LD: 0.9188
AF	LD	Non-LD: 0.9018; Gaussian noise: 0.9054	Final LD: 0.9197	Shuffled-label LD: 0.8974
CR	Band	Full 31 bands: 0.9238	Top-12 bands: 0.9221	Random 12 bands: 0.7815±0.0642; low-importance 12 bands: 0.6426
AF	Band	Full 31 bands: 0.9018	Top-12 bands: 0.8392	Random 12 bands: 0.7348±0.0556; low-importance 12 bands: 0.6253

Macro-F1 is reported for all settings. Gaussian-noise augmentation was used as a simple perturbation control. The shuffled-label LD control trained the LD module with randomly permuted class labels within each training fold. For the band-selection control, the selected top-12 bands were taken from the reduced-band results. Random-band results are reported as mean ± SD over 30 random 12-band subsets.

**Table 9:** Internal 70/30 validation and independent external validation of the selected sample-level models.

Dataset	Validation Setting	Model	Acc.	Pre.	Rec.	F1
CR	70/30 internal	Linear SVM	0.9188±0.0415	0.9281±0.0382	0.9188±0.0415	0.9176±0.0430
CR	70/30 internal	LD-augmented SVM	0.9375±0.0357	0.9440±0.0319	0.9375±0.0357	0.9364±0.0372
AF	70/30 internal	Linear SVM	0.8956±0.0231	0.8972±0.0224	0.8956±0.0231	0.8948±0.0235
AF	70/30 internal	LD-augmented SVM	0.9147±0.0194	0.9162±0.0187	0.9147±0.0194	0.9141±0.0198
CR	External, n=160	Linear SVM	0.9000	0.9068	0.9000	0.8989
CR	External, n=160	LD-augmented SVM	0.9250	0.9295	0.9250	0.9247
AF	External, n=200	Linear SVM	0.8800	0.8826	0.8798	0.8790
AF	External, n=200	LD-augmented SVM	0.9200	0.9224	0.9199	0.9195

Values for the 70/30 internal validation are reported as mean ± standard deviation over 30 repeated group-aware train/test splits. External validation values were calculated using newly collected independent samples. The external CR validation set contained 160 samples, with 40 samples per class, and the external AF validation set contained 200 samples, with 50 samples per geographical origin. External samples were not used for model training, parameter selection, LD fitting, wavelength selection, or any part of model optimization.

**Table 10:** Tentative spectral–chemical interpretation of informative LED-band regions.

Spectral Region	Representative LED Bands in This Study	Tentative Spectral-Chemical Relevance
Visible violet-blue region	365, 410, 420, 440, 455, 465, 475 nm	Surface color, browning-related optical variation, pigments, and phenolic/flavonoid-associated responses
Visible green-red region	500, 520, 535, 570, 585, 595, 620, 655, 680, 700, 730, 750 nm	Yellow-brown appearance, chromophore-related variation, tissue color difference, and matrix scattering
Short-wave NIR region	770, 805, 820, 850, 880, 900, 940, 980 nm	Broad O-H/C-H/N-H overtone or combination responses, moisture-related variation, and sample-matrix scattering

The interpretation is tentative because the present spectra were ROI-derived mean gray-value spectra rather than calibrated absorbance spectra or quantitative chemical measurements.

## Data Availability

The data presented in this study are available from the corresponding author upon reasonable request.

## Abbreviations

The following abbreviations are used in this manuscript:

AF	Aurantii Fructus
CR	Codonopsis Radix
LD	Lightweight diffusion
MLP	Multilayer perceptron
MSC	Multiplicative scatter correction
ROI	Region of interest
SG	Savitzky–Golay
SNV	Standard normal variate
SVM	Support vector machine
Vis/NIR	Visible and near-infrared
